# Long-term effects of thinning intensity on individual growth and stand basal area recovery in a mixed broadleaf-Korean pine forest

**DOI:** 10.3389/fpls.2026.1806543

**Published:** 2026-05-13

**Authors:** Yue Sun, Fushan Cheng, Luping Jiang, Ye Luo, Zhongqi Xu, Huaijiang He

**Affiliations:** 1 College of Forestry, Hebei Agricultural University, Baoding, China; 2 Jilin Province Degraded Forest Ecosystem Restoration and Reconstruction Interregional Cooperation Science and Technology Innovation Center, Changchun, China; 3 College of Forestry and Grassland Science, Jilin Agricultural University, Changchun, China; 4 Jilin Provincial Academy of Forestry Sciences, Changchun, China

**Keywords:** driving factors, individual tree growth, multi-species forest, Northeastern China, thinning

## Abstract

**Introduction:**

Selective thinning is a widely applied silvicultural practice aimed at reducing competition and accelerating the growth of target trees. However, excessive tree removal can lead to a significant short-term decline in stand-level productivity and delay structural recovery. Therefore, understanding the trade-offs between individual tree release and stand basal area recovery is crucial for sustainable forest management.

**Methods:**

Using 11 years of repeated inventory data from a mixed broadleaf–Korean pine forest in northeastern China, we examined tree growth and stand recovery under four thinning intensities (0%, 20%, 40%, and 60%). Principal component analysis (PCA) and hierarchical clustering were used to classify 19 tree species into three functional groups.

**Results:**

The results showed that thinning effects were time dependent. A growth lag phase was observed in the first post-thinning period (2011–2013), followed by a stronger growth response in later study periods, when the high-intensity treatment (60%) showed nearly double the individual growth rate of the control. However, species responses were asymmetric; shade-tolerant climax species (Cluster 3) exhibited the strongest competitive release, whereas pioneer species (Cluster 1) showed minimal sensitivity possibly because of species strategy and tree size. At the stand level, low-intensity thinning (20%) showed a compensatory growth trend, and the fitted linear trend suggested that stand basal area may approach or exceed the control level within about 16 years. In contrast, the high-intensity treatment showed a much slower projected recovery trajectory. The linear mixed-effects model showed that tree growth was mainly related to tree size and spatial structure. Variance decomposition indicated that individual-tree variables contributed the largest share of the explained variance (59.5%).

**Discussion:**

Under the conditions of this study, the 20% treatment plot showed the most balanced outcome between stand basal area recovery and individual tree growth during the observed period. These results support the use of structure-based silviculture in mixed broadleaf–Korean pine forests, especially by improving growing space for desirable Cluster 3 individuals while maintaining stand heterogeneity.

## 1 Introduction

Forests play a pivotal role in global carbon cycling and ecosystem service provision (Yang et al., 2023). However, forest degradation caused by climate change and unsustainable harvesting has increased the need for science-based management strategies that enhance both productivity and resilience (Hadden and Grelle, 2016; Mekonnen et al., 2017; Gatti et al., 2021; Hogan et al., 2024). Thinning, a fundamental silvicultural practice, is widely used to regulate stand density, redistribute resources such as light, water, and nutrients to residual trees, and improve stand structure (Bose et al., 2018). However, the ecological and production outcomes of thinning depend strongly on treatment intensity, intervention frequency, and the capacity of residual trees and stands to adjust after disturbance (Hu et al., 2020; Geng et al., 2021; Yano et al., 2021).

A long-standing challenge in thinning research is the trade-off between individual-tree growth release and stand basal area recovery (Riutta et al., 2018). High-intensity thinning can maximize the growth of residual individuals by reducing competitive pressure, but it may simultaneously create a state of understocking in which the remaining stand cannot fully occupy the available growing space, thereby slowing the recovery of stand structure. By contrast, low-intensity thinning may favor rapid stand closure but fail to trigger a marked growth release in target trees (Gibson et al., 2011; Riutta et al., 2021; Cabon et al., 2023). From the perspective of thinning, this trade-off is also consistent with the Intermediate Disturbance Hypothesis, in that moderate disturbance may balance competitive release with the retention of stand structural integrity, whereas very weak or very strong disturbance may reduce this balance (Molino and Sabatier, 2001).

Forest recovery is a multidimensional concept that may include biomass recovery, biodiversity recovery, compositional recovery, and structural recovery (Poorter et al., 2021). In the present study, however, we use the term recovery in a narrower operational sense, referring specifically to the post-thinning recovery of stand basal area. By contrast, responses at the individual-tree level are described as growth responses or growth release rather than recovery.

A critical, yet often overlooked, dimension of thinning research is the temporal trajectory of stand basal area recovery and tree growth response. The timing of post-thinning stand recovery and tree growth release provides useful insight into stand adjustment after disturbance (Huang and Asner, 2010; Bedrij et al., 2022). Understanding the temporal dynamics of this recovery—specifically, the “lag phase” immediately post-disturbance and the subsequent “compensatory growth”—is essential for determining sustainable harvest intervals (Riutta et al., 2021; Bartels and Macdonald, 2023). The lag phase is defined as the post-treatment period during which measurable growth enhancement is limited or absent because residual trees are still adjusting physiologically and structurally to the altered environment (Puettmann and Bauhus, 2023). Such lagged responses may arise from crown and foliage acclimation, reallocation of carbon to root systems, and the gradual reorganization of competitive interactions after canopy opening (Lehtonen et al., 2023). However, many previous studies are based on short-term observations (< 5 years), which may fail to capture delayed responses and long-term stand recovery trajectories. Previous studies suggest that post-thinning lag may last from several years to more than a decade, depending on species traits, stand structure, thinning intensity, and site conditions. For example, canopy trees in selection-harvested stands showed delayed growth responses, with maximum basal area increment occurring 3–15 years after harvest, and the magnitude of response differed among shade-tolerance groups and tree sizes (Jones et al., 2009). Likewise, long-term thinning studies have shown that treatment effects can remain evident for more than a decade, and that increased growth of residual trees does not necessarily translate into rapid stand-level recovery (Mahatara et al., 2025). This limitation is particularly important in temperate mixed forests, where contrasting life-history strategies among co-occurring species may result in asynchronous responses to thinning.

Thinning responses are rarely uniform across a stand because they are shaped by interactions among species strategy, individual tree size, competition, and spatial structure (Padilla-Martínez et al., 2024). Species differ in resource acquisition, shade tolerance, competitive ability, and life-history strategy, and these differences can be interpreted within the broader fast–slow spectrum of plant ecological strategies (Reich, 2014; Ford et al., 2025). As a result, tree species in mixed forests may exhibit strongly contrasting growth trajectories after canopy opening. At the same time, tree growth is also affected by individual size and the local competitive environment. While climatic factors influence growth at broader spatial scales (Maes et al., 2018; Wu et al., 2022), local biotic interactions often dominate at the stand and neighborhood scales (Tenzin et al., 2017). In natural mixed forests, high species richness and structural heterogeneity create asymmetrical competition (Larrieu et al., 2017; Croce et al., 2022; Pan et al., 2024; Soubeyrand et al., 2024). Trees with similar size may still differ in growth because their neighboring trees differ in size, species identity, and spatial arrangement. Likewise, stand structural attributes influence light interception, growing space, and the immediate competitive environment of residual trees (Buechling et al., 2017; Hui et al., 2019, Hui et al., 2023; Soubeyrand et al., 2024). Therefore, grouping species into functional cohorts based on biological traits and growth-related characteristics provides a useful framework both for ecological interpretation of species-specific thinning responses and for modeling growth dynamics in species-rich mixed forests (Tenzin et al., 2017; Padilla-Martínez et al., 2024).

Mixed broadleaf–Korean pine forest is an important climax forest type in northeastern China and has high ecological and conservation value. However, this forest type has also experienced long-term human disturbance and historical degradation, which makes the understanding of its post-thinning growth and recovery especially important for ecological restoration and sustainable management. Despite extensive research on thinning, an important gap remains in understanding how different functional groups in mixed temperate forests respond to thinning over the long term (e.g., >10 years), and how the relative importance of tree size, competition, and stand structure varies under different thinning intensities. In this study, we analyzed 11 years of repeated inventory data from a temperate mixed broadleaf–Korean pine forest. We used principal component analysis and hierarchical clustering to classify 19 tree taxa into functional cohorts, and then applied linear mixed-effects models to disentangle the drivers of individual tree growth. Specifically, we addressed the following questions: (1) How does thinning intensity differentially affect the growth rates of distinct species groups? (2) What are the temporal trajectories of stand basal area recovery under different thinning intensities? (3) How do individual tree attributes, competition, and stand structure jointly influence post-thinning tree growth?

## 2 Materials and methods

### 2.1 Study area

**Figure 1:**
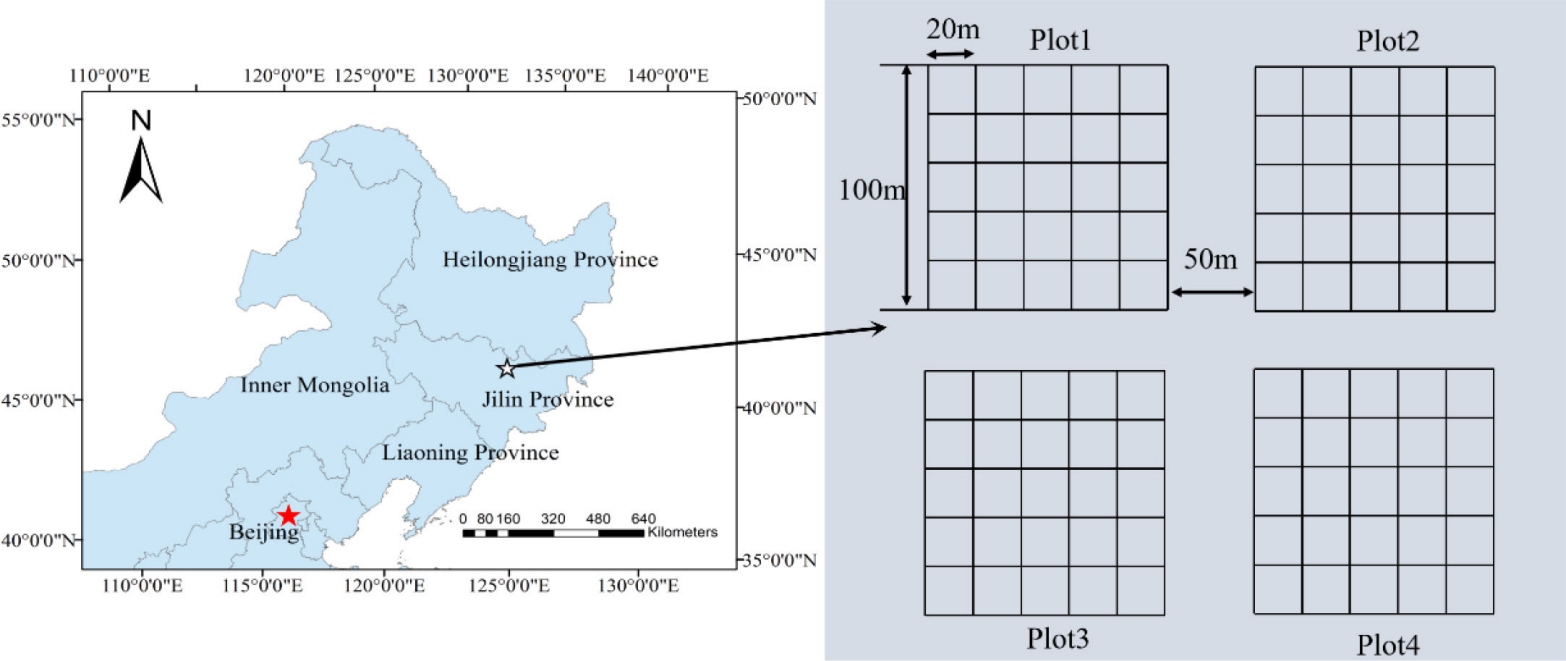
Location of the study area and schematic layout of the four 1-ha experimental plots. Plot 1, Plot 2, Plot 3, and Plot 4 correspond to the 0%, 20%, 40%, and 60% thinning treatments, respectively. Each plot (100 m × 100 m) was subdivided into 25 subplots of 20 m × 20 m. The distance between adjacent plots was greater than 50 m.

This study was conducted at the Jiaohe Forest Experimental Zone, Jilin Province, Northeastern China (43°57′31″ ~ 43°58′03″N, and 127°44′07″ ~ 127°44′40″E) (**Figure 1**). The prevailing climate in the area is a temperate continental mountainous one influenced by monsoon winds. The hottest month is July, with an average temperature of 21.7 °C, and the coldest month is January, with an average temperature of −18.6 °C. Precipitation is concentrated between June and August, with an average annual precipitation of 696 mm. The soil type was dark brown forest soil with a depth of > 60 cm. The vegetation belongs to the Changbai Mountain flora and the forest type is a broad-leaved Korean pine forest. The canopy was dominated by *Pinus koraiensis*, *Ulmus davidiana*, *Acer triflorum*, *Acer mandshuricum*, *Betula platyphylla*, *Quercus mongolica*, *Juglans mandshurica*, and *Fraxinus mandshurica*. Owing to various forest conservation policies in China, logging has been prohibited in the region for nearly 70 years (Hao et al., 2020).

### 2.2 Experimental design and thinning treatments

In July 2011, four permanent plots of 1 ha each (100 m × 100 m) were established in an area with relatively similar stand conditions in the Jiaohe Forest Experimental Zone. The distance between plots was greater than 50 m. The four plots were assigned to four thinning treatments with target basal area removal intensities of 0%, 20%, 40%, and 60%, respectively. During thinning, tree removal followed the target-tree silvicultural rules and was distributed across each treatment plot as evenly as possible to avoid highly concentrated openings. Each 1-ha plot was further divided into 25 subplots of 20 m × 20 m (0.04 ha), which were used as inventory units for repeated measurements and analysis. All trees with diameter at breast height (DBH) ≥ 5 cm in each subplot were measured. For each tree, species identity, DBH, crown width, and its spatial position within the subplot were recorded. Tree position was recorded as subplot-based coordinates to describe the relative spatial arrangement among neighboring trees. Pre-treatment comparisons showed no significant differences among the four 1-ha plots in stand DBH, stand density, basal area, or canopy cover (**Table 1**), indicating that the plots had similar stand conditions before thinning. The silvicultural treatments were carried out in December 2011 using a structure-based approach known as “Target Tree Silviculture”. The treatment aimed to release target trees with good stem quality and vitality while reducing local competition. The main marking rules were as follows: (1) removal of trees with pest damage, disease, or clear stem defects; (2) thinning of clustered individuals to reduce competition; and (3) retention of rare species to maintain tree species diversity. To reduce soil disturbance and damage to residual trees, harvested timber was extracted using low-impact animal skidding. Repeated investigations were conducted in 2013, 2015, 2018, and 2022 to monitor post-thinning growth and stand development. Thus, thinning was applied at the 1-ha plot level, whereas the 20 m × 20 m subplots served as repeated inventory units for within-plot analysis.

**Table 1:** Pre-treatment and post-treatment stand characteristics and site conditions of the four 1-ha plots under different thinning treatments.

Variable		Control (0%)	Low intensity (20%)	Moderate intensity (40%)	High intensity (60%)
Latitude		43°57.748’N	43°57.784’N	43°58.062’N	43°58.384’N
Longitude		127°43.888’E	127°44.388’E	127°44.317’E	127°45.532’E
Altitude (m)		453	443	430	447
Slope grade (°)		1	4	5	3
Aspect		NE	NE	NE	NE
DBH (cm)	pre-treatment	14.6	13.9	14.8	12.4
	post-treatment	14.6	13.8	14.8	12.7
Basal area (m^2^/ha)	pre-treatment	30.0	29.4	30.4	30.5
	post-treatment	30.0	24.4	19.8	14.7
Canopy cover	pre-treatment	0.9	0.9	0.9	0.9
	post-treatment	0.9	0.8	0.6	0.5

### 2.3 Relative growth rate

To compare the growth vitality of individual trees across different thinning intensities, we calculated the mean periodic relative growth rate (*BA_rate_*, Equations 1) using Pressler’s formula. Unlike simple relative growth rates based solely on initial size, Pressler’s formula provides a robust estimate of the mean annual growth percentage relative to the average basal area during the observation period. The formula is expressed as follows:

(1) $$BA_{rate}=\frac{BA_{t}-BA_{t-n}}{BA_{t}+BA_{t-n}}\times \frac{200}{n}$$

where *BA_rate_* represents the mean annual basal area growth percentage (%); *BA_t_* is the basal area of the tree at the end of the study period year *t*; *BA_t-n_* is the basal area at the beginning of the period year *t-n*; and *n* denotes the time interval (in years) between the two inventories.

### 2.4 Variable selection and measurement

We analyzed the drivers of tree growth by selecting Basal Area Increment (BAI) as the response variable. To explain the variance in BAI, we selected a total of 11 predictor variables categorized into four levels: individual tree characteristics, competition indices, stand structure, and species biological traits (**Table 2**). At the individual level, Diameter at Breast Height (DBH) was used as the primary proxy for tree size. Competition intensity was quantified using the Basal Area of Larger trees (BAL), which represents size-asymmetric competition. To capture the spatial heterogeneity of competition, we calculated three structural parameters: Mingling (M*_i_*), Uniform Angle Index (W*_i_*), and Dominance (U*_i_*). Furthermore, we employed the Comprehensive Forest Spatial Structure Index (FSS*_i_*) to provide a holistic evaluation of the stand’s structural complexity (Hui et al., 2019). Stand-level variables included stand density (N), quadratic mean diameter (D_g_), and dominant diameter (D_dom_). Additionally, a shade tolerance score (SST) was assigned to each species to account for functional trait differences in light acquisition strategies.

**Table 2:** Description of the response and predictor variables used in the growth models.

Category	Variables	Acronym	Unit	Description	Resolution
Response	Basal area increment	BAI	cm^2^/yr	Periodic annual increment of basal area	Individual
Predictors	Diameter at breast height	DBH	cm	Tree size at the beginning of the period	Individual
	Basal area of larger trees	BAL	cm^2^	Sum of basal area of trees larger than the subject tree	Competition variables
	Mingling	M*_i_*	unitless	Spatial isolation of species	Competition variables
	Uniform angle index	W*_i_*	unitless	Spatial distribution pattern of neighbors	Competition variables
	Dominance	U*_i_*	unitless	Size differentiation relative to neighbors	Competition variables
	Comprehensive index	FSS*_i_*	unitless	Forest Spatial Structure comprehensive index	Competition variables
	Quadratic mean diameter	D_g_	cm	Measure of the average tree size of the stand	Plot
	Stand density	N	trees·ha^-1^	Number of trees per hectare	Plot
	Dominant diameter	D_dom_	cm	The mean of the four trees with the largest DBH in the plot	Plot
	Species shade tolerance	SST^*^	score	The capacity for growth in the shade.	Species

^*^ The five-level score used for shade tolerance (0-1, very intolerant; 1-2, intolerant; 2-3, moderately tolerant; 3-4, tolerant; 4-5, very tolerant. See **Supplementary Table 1**).

### 2.5 Spatial structure indices

To comprehensively quantify the micro-environmental interactions, we utilized the Forest Spatial Structure Index (FSS*_i_*), which integrates three fundamental structural parameters based on the relationship between a reference tree *i* and its four nearest neighbors (n=4): Mingling (M*_i_*, Equation 2), Uniform Angle Index (W*_i_*, Equation 3), and Dominance (U*_i_*, Equation 4) (von Gadow and Hui, 2002).

Mingling (M*_i_*) describes the degree of spatial segregation among species. It is defined as the proportion of the four nearest neighbors that belong to a different species than the reference tree:

(2) $$M_{i}=\frac{1}{n}\sum_{j=1}^{n}v_{ij}$$

where *v_ij_* = 1 if neighbor *j* is a different species from reference tree *i*, and *v_ij_* = 0 otherwise.

Uniform Angle Index (W*_i_*) characterizes the horizontal distribution pattern of trees. It is calculated based on the angle between two adjacent neighbors and the reference tree:

(3) $$W_{i}=\frac{1}{n}\sum_{j=1}^{n}z_{ij}$$

where *z_ij_* = 1 if the angle is smaller than the standard angle α=72°, and *z_ij_* = 0 otherwise. A W*_i_* value within the interval [0.475, 0.517] indicates a random distribution, which is generally considered ideal for natural forests (Zhang et al., 2018).

Dominance (U*_i_*) reflects the size differentiation and competitive status of the reference tree relative to its neighbors:

(4) $$U_{i}=\frac{1}{n}\sum_{j=1}^{n}k_{ij}$$

where *k_ij_* = 1 if the DBH of neighbor *j* is greater than that of reference tree *i*, and *k_ij_* = 0 otherwise. A lower U*_i_* indicates that the reference tree is dominant (larger than its neighbors), while a higher U*_i_* suggests suppression.

To synthesize these aspects, we calculated the Comprehensive Forest Spatial Structure Index (FSS*_i_*, Equation 5). Since raw values of M*_i_*, W*_i_*, U*_i_* have different ecological implications (e.g., high U*_i_* is negative for individual dominance, while high M*_i_* is positive for diversity), we first normalized these parameters into standardized scores (R*_k_*) ranging from 0 to 1 (**Table 3**). The FSS*_i_* was then derived using the unit circle method (Hui et al., 2023):

**Table 3:** Comparison of mean and standardized values of structural parameters.

Mean values of structure parameters	Mingling (*Mi*)	Uniform angle index (*Wi*)			Dominance (*Ui*)				
		*Wi* < 0.475	0.475≤*Wi*≥0.517	*Wi >*0.517	*Ui* < 0.47	0.47≤*Ui*≥0.9	0.49< *Ui >*0.51	0.51≤*Ui*≥0.53	*Ui >*0.53
Standardized values	Measured value	Measured value	1.0	0.5	1.0	0.75	0.5	0.25	0.0

(5) $$FSS_{i}=\frac{1}{m}\sum_{k=1}^{m}R_{k}^{2}$$

where *m* = 3 (the number of indices) and R*_k_* represents the standardized value of M*_i_*, W*_i_* and U*_i_*.

### 2.6 Plot variables and species classification

Plot-level characteristics included stand density (N) and quadratic mean diameter (D*_g_*, Equation 6), calculated as:

(6) $$D_{g}=\sqrt{\frac{1}{N}\sum_{i=1}^{N}d_{i}^{2}}$$

where d*_i_* is the DBH of the *i*-th tree. Additionally, the dominant diameter (D*_dom_*) was calculated as the mean DBH of the largest trees per unit area. Following the standard protocol (one dominant tree per 100m^2^), D*_dom_* in our 400 m^2^ plots represented the mean DBH of the four largest individuals. To facilitate functional group analysis, tree species were classified based on shade tolerance traits ($SST$). Tolerance values were assigned following Niinemets and Valladares (2006), with missing values imputed using data from congeneric species in similar temperate regions (**Supplementary Table 2**).

### 2.7 Functional grouping of tree species

The study area hosts a high diversity of woody plants, comprising 19 distinct tree taxa. Analyzing such a large number of species individually is statistically challenging due to limited sample sizes for rare species, which can introduce significant uncertainty (Ishihara et al., 2015; Padilla-Martínez et al., 2024). To address this, we employed a two-step multivariate approach—Principal Component Analysis (PCA) followed by Hierarchical Cluster Analysis (HCA)—to categorize species into functional groups (clusters) based on shared biological and structural attributes.

### 2.8 Statistical analysis

To reduce the effects of different measurement scales, all 11 variables (**Table 2**) were standardized using Z-score normalization. Principal component analysis (PCA) was then performed using the PCA() function in the FactoMineR package (Lê et al., 2008) in R 4.4.0 (R Core Team, 2024) to reduce dimensionality and multicollinearity. Based on the scores of the retained principal components, hierarchical cluster analysis was conducted using Ward’s minimum variance method. The number of clusters was selected by considering both clustering performance and ecological interpretability.

Differences in individual tree growth rates (BArate) among thinning treatments, study periods, and species groups were examined descriptively and statistically to compare post-thinning growth patterns. Changes in stand basal area over time under different thinning treatments were evaluated using linear regression.

To quantify the drivers of individual tree growth (BAI), we developed linear mixed-effects models (LMMs). Before model fitting, multicollinearity among predictor variables was assessed using the variance inflation factor (VIF) in the performance package (Lüdecke, 2021), and variables with VIF > 5 were excluded. Based on previous growth modeling studies (Wu et al., 2022), the full model was defined as:


$$ln(BAI_{ijk})=\beta_{0}+\beta X_{ijk}+\gamma DBH_{ijk}^{3}+\mu_{j}+v_{k}+\epsilon_{ijk}$$

where $BAI_{ijk}$ represents the basal area increment of tree *i* in subplot *j* at year *k*; $\beta_{0}$ is the intercept; $X_{ijk}$ is the vector of fixed effects, including individual variables (DBH), competition indices (BAL, FSS*_i_*), and stand characteristics (D*_g_*, N, D*_dom_*); and β is the vector of corresponding coefficients. To capture potential non-linear size effects, a cubic term for diameter (DBH^3^) was included γ. Random effects included Subplot $\mu_{j}$ and Year $v_{k}$ to account for nested sampling and temporal autocorrelation.

Because thinning treatment was applied at the 1-ha plot level, whereas the 20 m × 20 m subplots were used as repeated inventory units, differences among thinning treatments were interpreted cautiously, with emphasis on tree-level responses, within-plot variation, and temporal growth patterns. Model performance was evaluated using Akaike information criterion (AIC), root mean square error (RMSE), and mean absolute error (MAE). Model stability was further assessed using 10-fold cross-validation. The relative importance of predictors in the final BAI model was quantified by variance decomposition of the explained variance using the *rdacca.hp* package in R (Lai et al., 2022).

## 3 Results

### 3.1 Functional grouping of tree species

**Figure 2:**
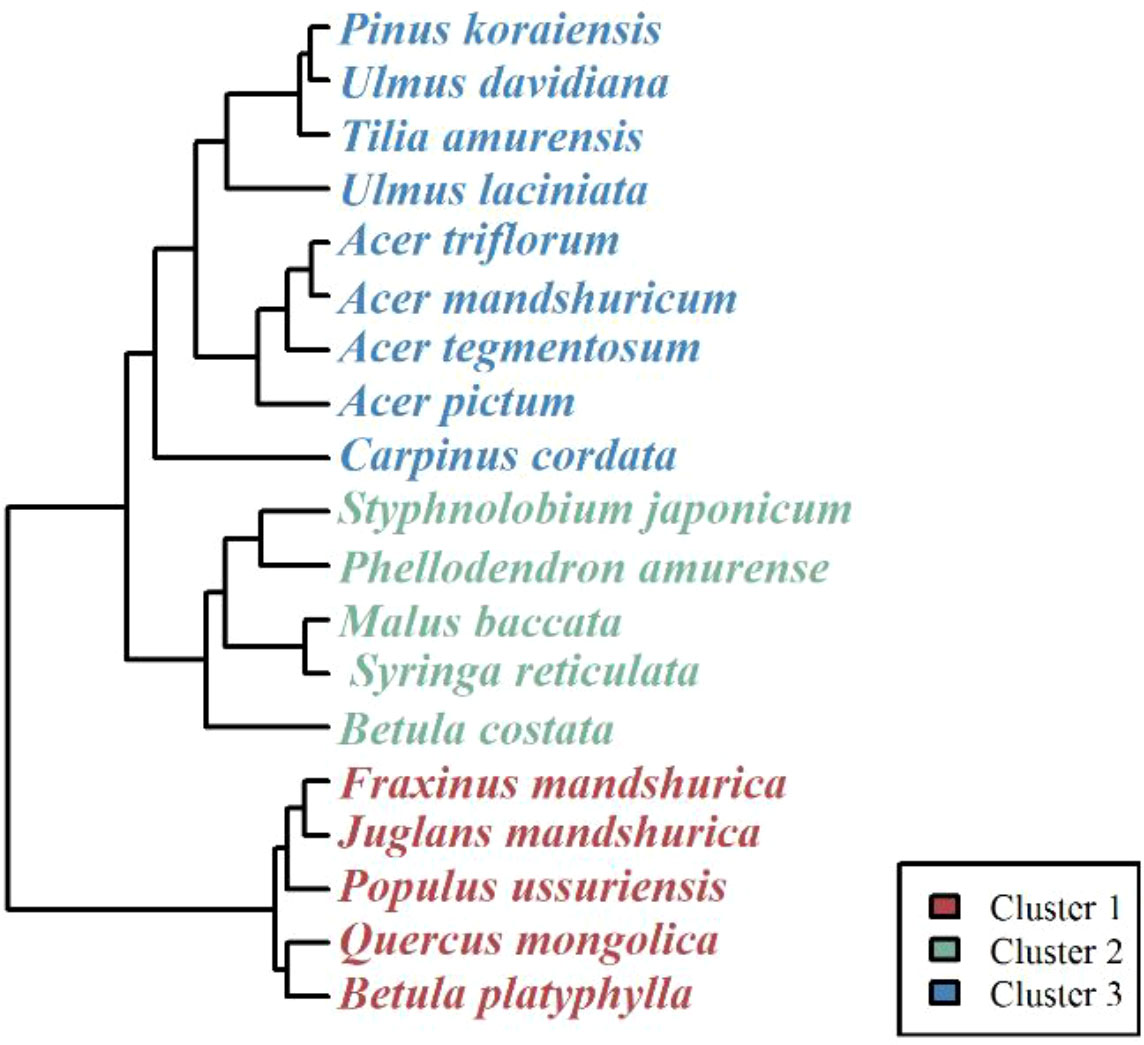
Dendrograms of the three tree species clusters based on the first five PCs. Different colors represent different clusters.

Principal Component Analysis (PCA) was performed on the 11 selected functional and structural variables to reduce data dimensionality. The first five principal components (eigenvalues > 1) were retained, cumulatively explaining 71.06% of the total variance. To determine the optimal number of functional groups, we evaluated the clustering validity indices (**Supplementary Figure 1**). The silhouette analysis indicated stable clustering structures for *k* values ranging from 2 to 8, with silhouette coefficients between 0.35 and 0.39 (**Supplementary Figure 1a**). Although higher *k* values yielded marginally better silhouette scores, we selected a three-cluster solution (*k* = 3) to balance statistical performance with ecological interpretability and to ensure sufficient sample sizes within each group for subsequent growth modeling. The three-cluster model explained 26.73 of the variance (**Supplementary Figure 1c**), accounting for 58.17% of the total variability (**Supplementary Figure 1d**), indicating that the classification effectively captured the differentiation among species. Based on the hierarchical clustering dendrogram (**Figure 2**), the 19 tree taxa were categorized as follows: Cluster 1 (5 species): Comprised fast-growing or canopy-dominant broadleaf species, including *Fraxinus mandshurica*, *Juglans mandshurica*, *Populus ussuriensis*, *Quercus mongolica*, and *Betula platyphylla*. Cluster 2 (5 species): Consisted of *Styphnolobium japonicum*, *Phellodendron amurense*, *Malus baccata*, *Syringa reticulata*, and *Betula costata*. Cluster 3 (9 species): The largest group, containing the climax conifer *Pinus koraiensis* along with shade-tolerant broadleaves such as *Tilia amurensis*, *Ulmus* spp., *Carpinus cordata*, and various *Acer* species (*A. triflorum*, *A. mandshuricum*, *A. tegmentosum*, *A. pictum*).

### 3.2 Tree growth responses under different thinning treatments and species groups

**Figure 3:**
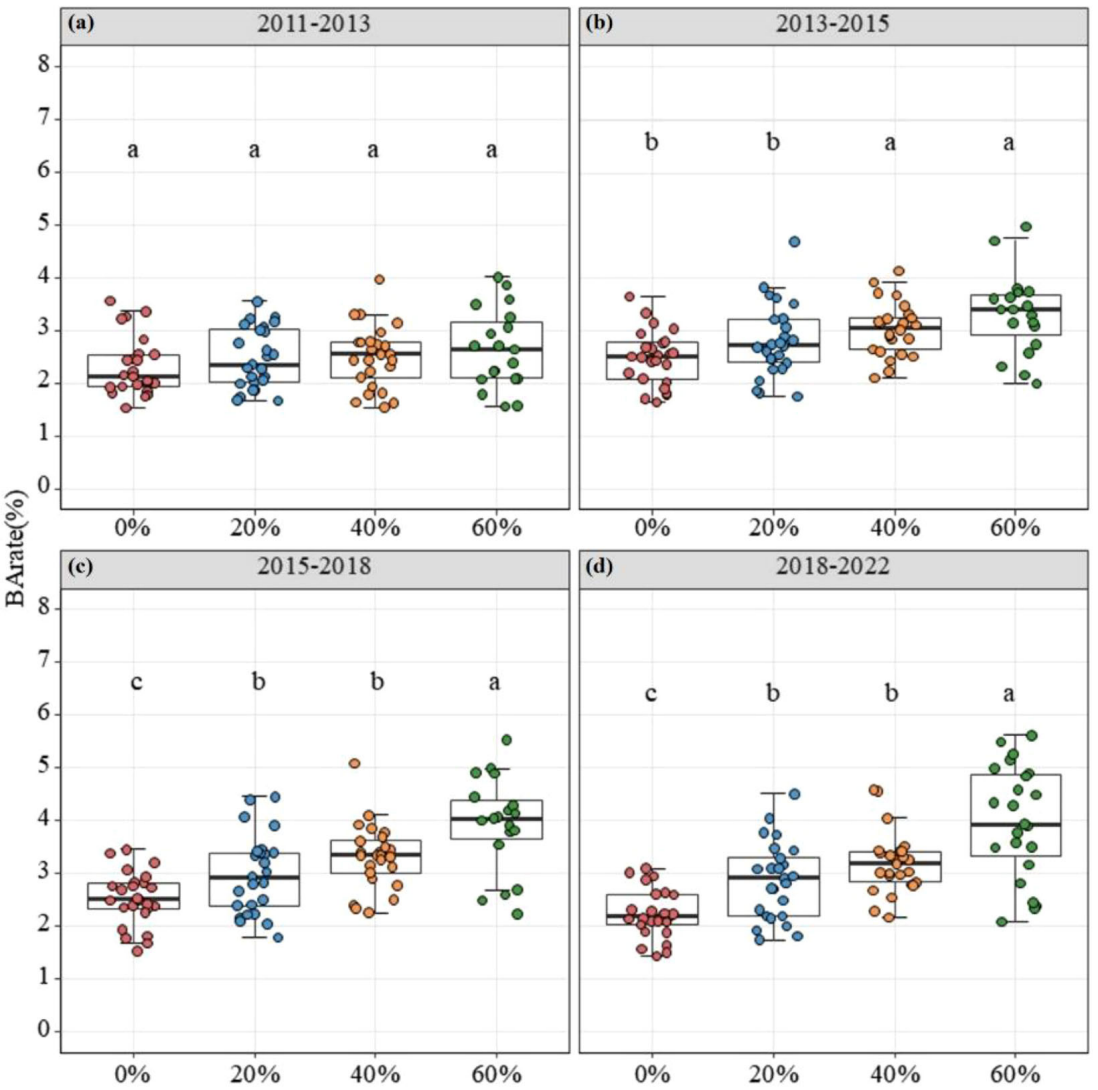
Changes in tree BArate across different thinning intensities during various recovery stages: **(A)** 2011-2013, **(B)** 2013-2015, **(C)** 2015-2018, and **(D)** 2018-2022. Different lowercase letters indicate significant differences among thinning treatments within the same study period at *p* < 0.05.

Individual tree growth rates (BA*_rate_*) showed clear differences among thinning treatments over time (**Figure 3**). In the initial post-thinning period (2011–2013), no significant differences were observed among thinning intensities, with BA*_rate_* ranging from 2.2% to 2.6% across all plots (**Figure 3a**). However, as stand development progressed, growth trajectories gradually diverged among treatments, and the plots with higher thinning intensity generally showed faster tree growth in the later study periods, although this advantage was not uniform across all study intervals (**Figures 3b–d**). By the 11th year post-thinning (2018–2022), trees in the high-intensity plots (60%) exhibited the highest growth rate (3.99 ± 2.40%), which was nearly double that of trees in the control plots (2.22 ± 1.60%). Longitudinal analysis further revealed distinct recovery patterns across treatments (**Supplementary Figure 2**). In the Control (0%) and Low-intensity (20%) plots, tree growth rates remained relatively stable over the study period, showing no statistically significant temporal trend (**Supplementary Figures 2a, b**). In contrast, trees in the Moderate (40%) and High-intensity (60%) plots exhibited a continuous and significant acceleration in growth rates over time (**Supplementary Figures 2c, d**), indicating a sustained release effect from competition.

**Figure 4:**
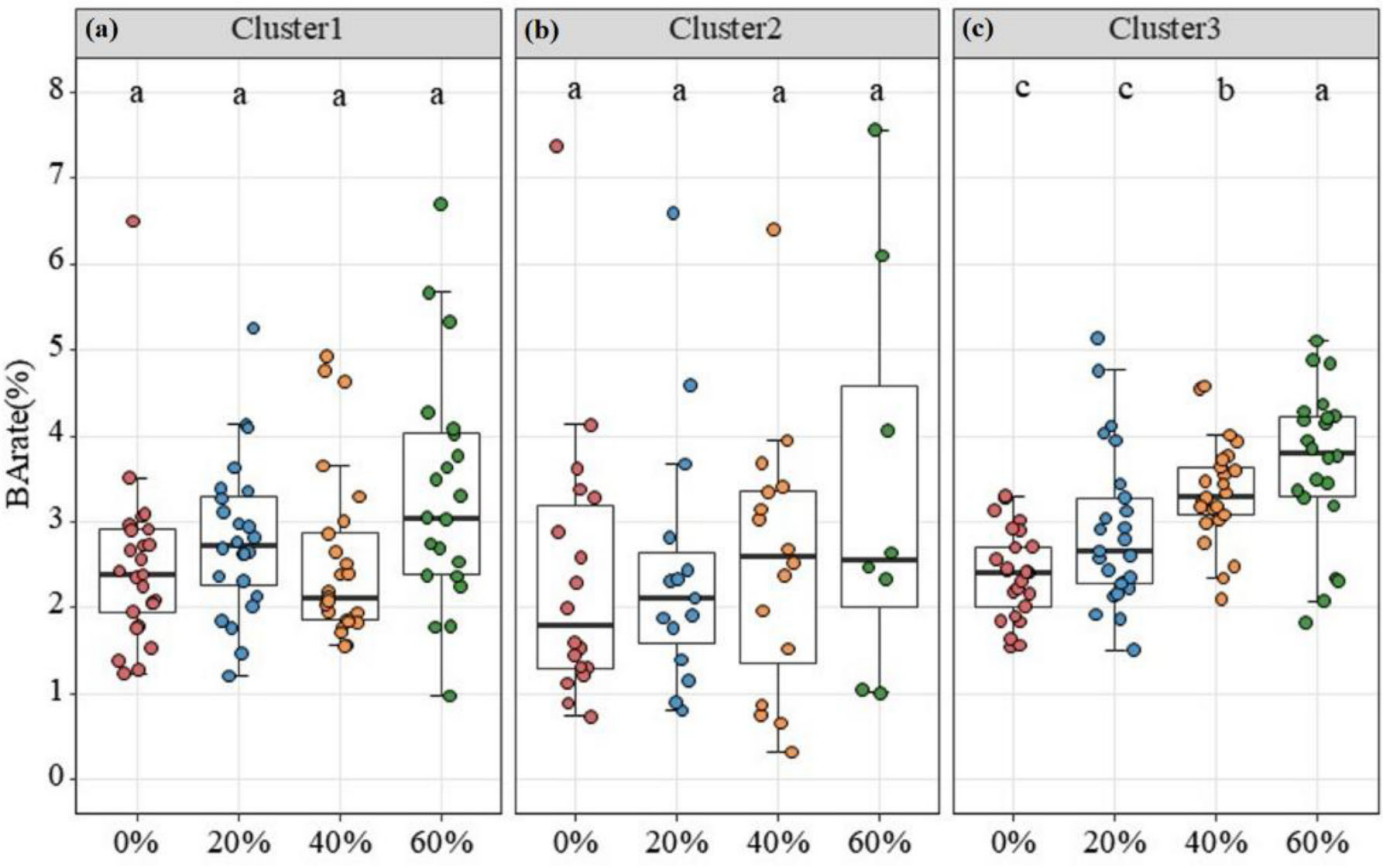
Tree BArate of the three species groups under different thinning treatments: **(A)** Cluster 1, **(B)** Cluster 2, and **(C)** Cluster 3. Different lowercase letters indicate significant differences among treatments within the same species group at *p* < 0.05.

Tree growth patterns differed among the three functional clusters under the four thinning treatments (**Figure 4**). For Cluster 1 and Cluster 2, BA*_rate_* did not show clear statistical differences among thinning treatments (*p*>0.05; **Figures 4a, b**). By contrast, Cluster 3 showed the clearest increase in growth rate across the thinning treatments (**Figure 4c**). The growth rate of Cluster 3 increased linearly with thinning intensity, although no significant difference was observed between the Control (0%) and Low-intensity (20%) treatments. This indicates that Cluster 3 species (primarily shade-tolerant and climax species) require moderate to high disturbance intensities to trigger a significant growth release.

### 3.3 Stand basal area recovery under different thinning treatments

**Figure 5:**
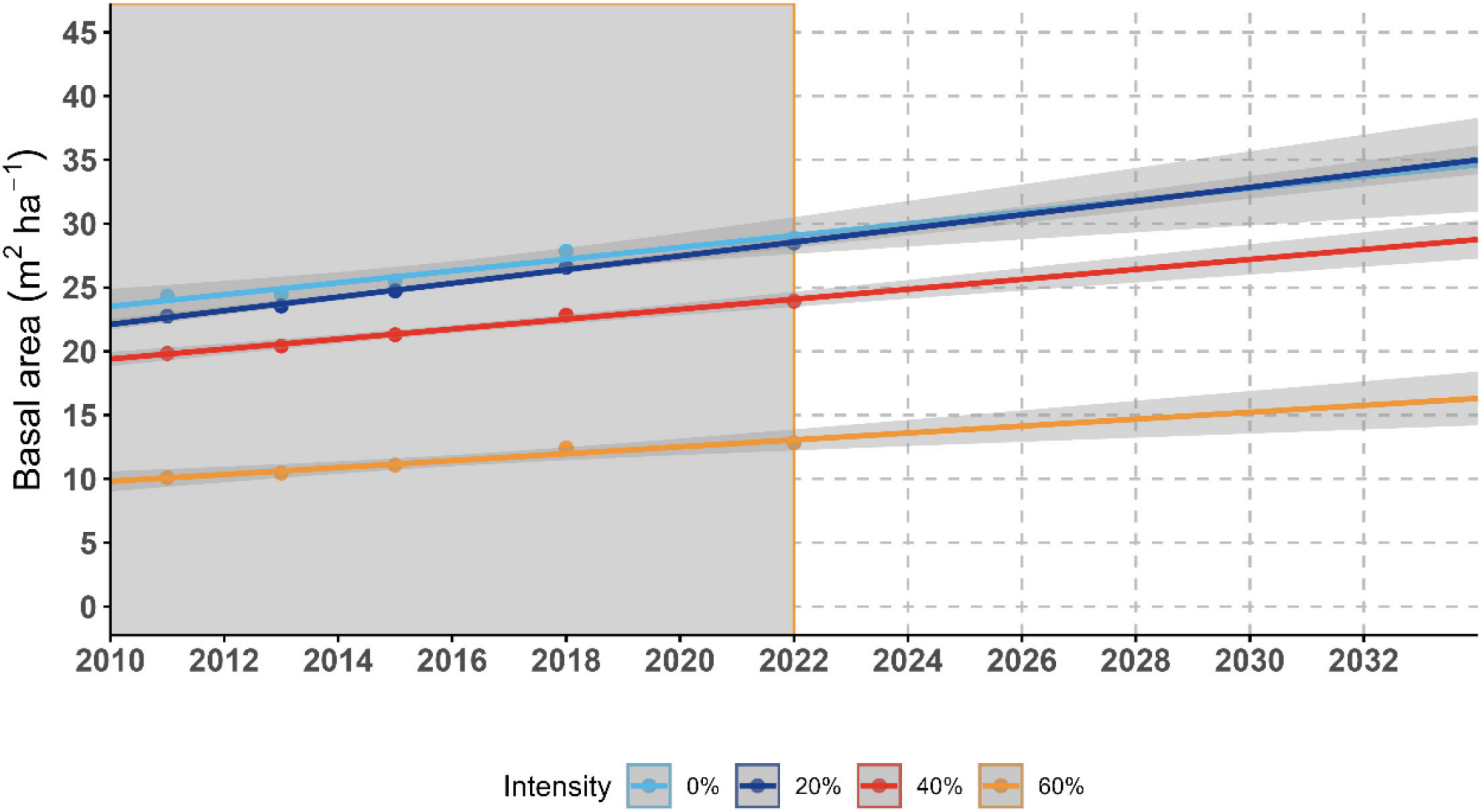
Linear regression of the average basal area for each thinning intensity plot from 2011 to 2022. Notes: the gray area represents the observation period, and the white area represents the extrapolated period based on the fitted linear trend.

Linear regression of stand basal area showed different recovery trajectories among the four thinning treatment plots during the 11-year monitoring period (2011–2022) and in the projected period (**Figure 5**). For the Low-intensity treatment (20%), stand basal area exhibited a rapid recovery. By 2022, stand basal area in the low-intensity treatment plot had already exceeded the pre-thinning baseline, and the fitted linear trend suggested that it may approach or exceed the control level within about 16 years after thinning. This pattern suggests that the low-intensity treatment plot had a relatively strong compensatory growth trend during the observation period. If the observed trends continue, the moderate-intensity treatment plot may return to the pre-thinning basal area level within the next few years. In contrast, the high-intensity treatment plot showed a much slower projected recovery trajectory, with a substantially longer time required to regain pre-treatment basal area, despite showing the highest overall annualized increase during 2011–2022, likely because the larger initial reduction in basal area required a longer time to recover to the pre-thinning baseline.

To further compare temporal changes in stand recovery, we summarized the annual percentage change in mean stand basal area across study periods for the four thinning treatments (**Table 4**). The results showed that the high-intensity treatment plot had the highest overall annualized increase from 2011 to 2022 (3.31 ± 1.39% per year), followed by the low-intensity (2.16 ± 0.57% per year), moderate-intensity (2.10 ± 0.72% per year), and control plots (1.82 ± 0.33% per year). However, the high-intensity treatment also showed the largest among-subplot variation, especially during 2015–2018 (4.72 ± 2.88% per year). Although the high-intensity treatment plot showed the highest relative increase in stand basal area (**Table 4**), its absolute stand basal area recovery remained slower than that of the low-intensity treatment plot during the observed period (**Figure 5**).

**Table 4:** Annual percentage change in stand basal area across study periods under different thinning treatments (mean ± SD).

Thinning treatment	2011–2013 (% per year)	2013–2015 (% per year)	2015–2018 (% per year)	2018–2022 (% per year)	2011–2022 (% per year)
Control (0%)	1.55 ± 0.44	2.25 ± 0.56	2.16 ± 0.46	1.49 ± 0.29	1.82 ± 0.33
Low (20%)	1.83 ± 0.67	2.61 ± 0.79	2.54 ± 0.72	1.82 ± 0.63	2.16 ± 0.57
Moderate (40%)	1.55 ± 0.47	2.31 ± 0.80	2.54 ± 1.19	1.94 ± 0.76	2.10 ± 0.72
High (60%)	2.03 ± 1.11	3.30 ± 1.61	4.72 ± 2.88	2.63 ± 1.28	3.31 ± 1.39

Values represent annualized percentage change in stand basal area across the 25 subplots within each treatment plot and are presented as mean ± SD for each study period.

### 3.4 Drivers of individual tree basal area increment

**Figure 6:**
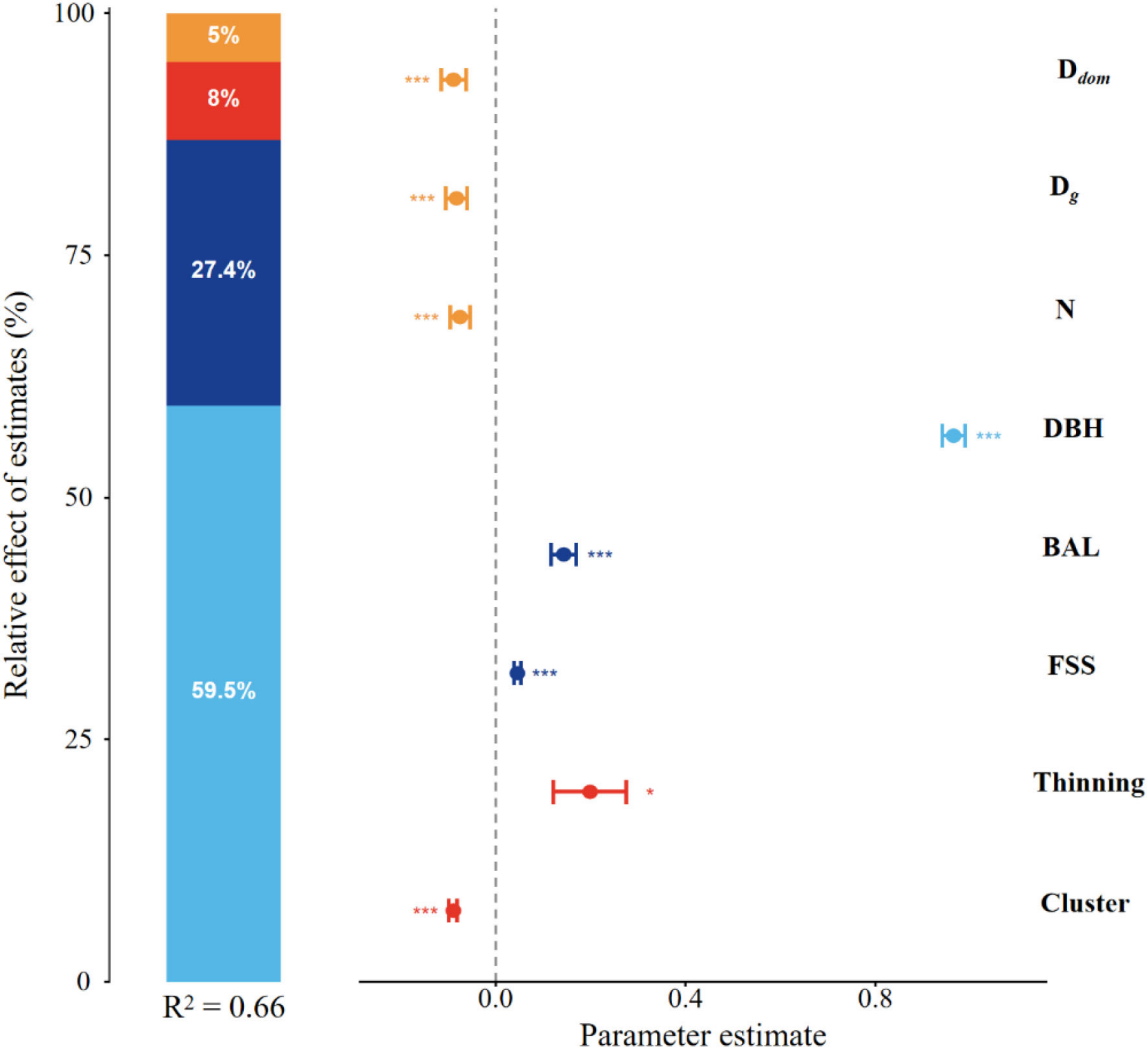
Relative effects of multiple variables of BAI. Standardized coefficients of the predictors are shown with 95% confidence intervals. Relative importance was estimated by variance decomposition of the explained variance. DBH, diameter at breast height; BAL, basal area of larger trees; FSS, comprehensive forest spatial structure index; D*_g_*, quadratic mean diameter; N, stand density; D*_dom_*, dominant diameter. Blue bars indicate individual-tree variables, dark blue bars indicate competition variables, red bars indicate categorical variables, and orange bars indicate stand variables. *p < 0.05; **p < 0.01; ***p < 0.001.

The linear mixed-effects model for basal area increment (BAI) showed good agreement between predicted and observed values (RMSE = 0.53; MAE = 0.42; **Supplementary Figures 3**, **4**). The final model explained 66% of the total variance in BAI (R^2^ = 0.66). The results shown in **Figure 6** were derived from this final BAI model.

Variance decomposition of the final BAI model showed that individual-tree variables had the largest contribution to the explained variance (59.5%), followed by competition variables (27.4%), thinning treatment (8.0%), and stand variables (5.0%). Among the predictors, DBH showed the strongest positive effect on BAI. D*_g_*, N, and D*_dom_* showed negative effects on individual tree growth. BAL and FSS were positively related to BAI. BAI also differed among thinning treatments, with higher-intensity treatment plots generally showing greater tree-level growth. In addition, significant differences were observed among species groups, indicating that functional identity was an important source of variation in growth.

## 4 Discussion

### 4.1 Changes in individual tree growth rates among species groups and intensities

Thinning alters forest structure by opening the canopy, reducing competition among trees, and increasing growing space, which may lead to different growth responses among species with different ecological traits (Condé et al., 2022). Our results showed a clear temporal pattern in post-thinning growth. In the first post-thinning period (2011–2013), tree growth rates did not differ strongly among thinning treatments (**Figure 3a**). This suggests that an immediate growth release did not occur after thinning. One possible reason is that residual trees needed time to adjust to the new light and microclimatic conditions after canopy opening (Jiao et al., 2021). Such adjustment may include crown and foliage acclimation, changes in water demand, and shifts in carbon allocation (Freschet et al., 2021; Lehtonen et al., 2023). However, as the stands continued to develop after thinning, growth trajectories gradually diverged among treatments, and the higher-intensity treatment plots generally showed greater tree growth in the later study periods (**Figures 3b–d**), indicating a delayed release effect (Sullivan et al., 2022; Puettmann and Bauhus, 2023).

Natural forests are complex systems with many coexisting species, and tree growth patterns are therefore not uniform (Padilla-Martínez et al., 2024). In our study, the three functional groups showed different growth responses to thinning. Cluster 1 consisted mainly of pioneer or light-demanding species, such as *Betula platyphylla* and *Populus ussuriensis*. However, these species did not show a strong growth increase after thinning. Light-demanding species are not always the first to benefit from canopy opening, because they already require relatively high light conditions for survival and growth (Orellana and Vanclay, 2018). Therefore, a sudden increase in light may not always produce an immediate growth advantage. In addition, tree size may also have affected the response (Mehtätalo et al., 2013). The mean DBH of Cluster 1 was 26.1 cm, whereas the mean DBH values of Cluster 2 and Cluster 3 were 12.4 cm and 17.4 cm, respectively (**Supplementary Figure 5**). These values suggest that growth response should be interpreted by considering both species strategy and tree size. A further possible explanation is that pioneer species may not allocate newly available resources directly to stem growth after thinning. Instead, part of the additional carbon may be used for reproduction, root expansion, or other structural adjustment (Adler et al., 2014; Ford et al., 2025). In contrast, Cluster 3, which included *Pinus koraiensis* and several shade-tolerant broadleaved species, showed a stronger increase in growth across the thinning treatments (**Figure 3c**). This pattern may reflect the ability of later-successional and shade-tolerant species to persist under suppression and then respond to reduced competition after thinning (Ryu et al., 2024). At the same time, the interpretation of Clusters 1 and 2 should remain cautious, because these groups showed high within-group variation and smaller, more uneven sample sizes across treatments than Cluster 3 (**Supplementary Table 2**). Therefore, the differences among clusters should not be interpreted as a simple contrast between light demand and shade tolerance, but rather as the joint result of species strategy, tree size, and post-thinning adjustment (Padilla-Martínez et al., 2024).

### 4.2 Recovery dynamics of basal area following different thinning intensities

Our results showed a clear trade-off between individual tree growth release, relative stand basal area increase, and absolute stand basal area recovery. These metrics do not describe exactly the same management outcome. Relative basal area increment reflects the strength of post-thinning growth release, whereas absolute stand basal area recovery reflects how quickly the stand compensates for the basal area removed by thinning and returns toward its pre-thinning structural condition.

In this study, the high-intensity treatment plot showed the highest relative increase in stand basal area (**Table 4**), indicating a strong post-thinning growth response. However, this treatment also started from the lowest post-thinning basal area. As a result, its higher relative growth was not sufficient to compensate for the much larger initial basal area reduction during the 11-year observation period. By contrast, the low-intensity treatment plot showed a lower relative increase, but it maintained a higher residual stocking after thinning and therefore showed a faster return toward pre-thinning and control-level basal area (**Figure 5**). This difference between growth release and structural recovery is consistent with previous studies showing that thinning or logging can enhance residual-tree growth while stand-level recovery of structure, biomass, or timber stock may remain slower and metric-dependent (Bose et al., 2018; Riutta et al., 2018).

From an ecosystem management perspective, such a long recovery cycle under high-intensity thinning may still present potential risks, including prolonged understocking, slower structural recovery, and a longer deficit in stand-level carbon storage and structural complexity (Roopsind et al., 2017; Bedrij et al., 2022). However, stand basal area recovery should not be used as the only criterion for judging whether one thinning treatment is better than another. Our study focused on stand basal area recovery and tree growth after thinning, and did not quantify other important dimensions of forest management, such as harvested biomass use, long-term carbon storage in wood products, belowground carbon, deadwood, or fire risk (Roopsind et al., 2017; Spinelli et al., 2019; Gundale et al., 2024).

Therefore, the management implication in our study is not based on projected total basal area alone, nor on relative basal area increment alone, but on their joint interpretation. Under the conditions of this study, the low-intensity treatment appeared more suitable when the management priority was relatively rapid stand structural recovery while maintaining some individual-tree growth release. In contrast, high-intensity thinning may still be appropriate when stronger release of residual trees is the primary objective.

### 4.3 The factors influencing the growth rate of individual trees.

In this study, the linear mixed-effects model explained 66% of the total variance in basal area increment (BAI) (**Figure 6**). Variance decomposition further showed that individual-tree variables made the largest contribution to the explained variance (59.5%), followed by competition variables (27.4%), thinning treatment (8.0%), and stand variables (5.0%). These results suggest that post-thinning tree growth in this mixed forest was mainly related to tree size and local competitive conditions. Among all predictors, DBH showed the strongest positive relationship with BAI, indicating that tree size was the main factor related to post-thinning growth. This result is consistent with previous studies showing that larger trees often have a stronger ability to acquire light and soil resources (Cienciala et al., 2016; Maes et al., 2018; Wu et al., 2022). However, our model also captured a non-linear relationship, where the growth efficiency of trees tends to decline after reaching a certain size threshold. This pattern suggests that while large trees are competitive, their growth is eventually limited by physiological constraints (e.g., aging or hydraulic resistance) and mechanical stability requirements (Forrester, 2021; Padilla-Martínez et al., 2024). At the stand level, stand density (N) exerted significant negative effects on individual growth. This result aligns with the fundamental principle of density-dependent regulation: in denser stands, the available resources per tree decrease, intensifying competition and suppressing individual vitality (Brunner and Forrester, 2020). This negative correlation provides the biological justification for thinning, as the artificial removal of competitors effectively increases the resource availability for the remaining trees. Additionally, the negative effects of quadratic mean diameter (D*_g_*) and dominant diameter (D*_dom_*) on BAI further confirm that in stands with higher competition, large trees may monopolize resources, suppressing the growth of other trees (Vandekerkhove et al., 2018). This supports the view that thinning can reduce stand competition and may help release resources for individual-tree growth (Fien et al., 2019).

Stand spatial structure comprehensive evaluation index (FSS) is significantly positively correlated with basal area increment (BAI), highlighting the importance of stand structure optimization in forest management. As a metric quantifying stand uniformity and spatial configuration, the positive effect of FSS may stem from balanced resource allocation and reduced competitive pressure (Wu et al., 2022; Padilla-Martínez et al., 2024). Additionally, the positive influence of the basal area of larger trees (BAL) suggests that while large trees dominate in resource competition, they may also indirectly promote the growth of other trees by improving light availability and stand structure (Looney et al., 2018). Finally, the significant differences among Species Clusters confirm that intrinsic biological traits determine growth potential. Even under similar environmental conditions, shade-tolerant climax species (Cluster 3) exhibited different growth strategies compared to pioneers (Roopsind et al., 2017). These results support the use of structure-based silviculture in mixed forests, because tree growth was related not only to stand density but also to tree size, local competition, and spatial structure. For species that appeared more sensitive to competition (e.g., Cluster 3), management may benefit from considering their immediate spatial neighborhood—ensuring they have adequate space and a favorable mix of neighbors—to maximize their growth potential (Schütz et al., 2016).

## 5 Conclusions

Our 11-year study highlights a clear trade-off between individual tree growth release and stand basal area recovery. The high-intensity thinning treatment showed the strongest tree growth release, but it was also associated with a much slower recovery of stand basal area. In contrast, the low-intensity thinning treatment (20%) showed a compensatory growth trend, with stand basal area projected to recover within about 16 years. Under the conditions of this study, the 20% treatment plot appeared more suitable when the management goal was to balance relatively rapid stand basal area recovery with individual tree growth during the observed period. Mixed-effects modeling showed that individual tree growth was mainly related to tree size and spatial structure. Tree growth responses also differed among species groups: shade-tolerant climax species (Cluster 3) showed a stronger response to competitive release, whereas pioneer groups (Clusters 1 and 2) were less sensitive, possibly because of differences in species strategy, tree size, and post-thinning allocation patterns. These results support the value of structure-based silviculture in mixed broadleaf–Korean pine forests. In this study, structure-based silviculture means that thinning should consider not only stand density, but also the size, spatial position, and neighboring conditions of individual trees. In mixed forests, this may be implemented by preferentially releasing high-value or desirable Cluster 3 individuals, while maintaining species mixture and avoiding excessive simplification of stand structure. In this way, thinning can be used to improve growing space for selected trees while still retaining structural and compositional heterogeneity at the stand level. Longer-term monitoring is needed to better evaluate whether these post-thinning growth and stand basal area recovery patterns remain stable over time.

## Data Availability

The raw data supporting the conclusions of this article will be made available by the authors, without undue reservation.
