# Supplementary material for: Long-term effects of thinning intensity on individual growth and stand basal area recovery in a mixed broadleaf-Korean pine forest
Source: Front Plant Sci. 2026 May 13;17:1806543. doi: 10.3389/fpls.2026.1806543 (PMC13212279; doi:10.3389/fpls.2026.1806543)
Supplement: Supplementary file 1 [file Table1.docx]

**Long-term effects of thinning intensity on individual growth and stand basal area recovery in a mixed broadleaf-Korean pine forest**

**Table S1** Biological characteristics of tree species in each cluster.

| Cluster | Species | Species shade tolerance | Characteristics |
| --- | --- | --- | --- |
| 3 | *Pinus koraiensis* | 3.83 | Evergreen coniferous trees, moderately shade-tolerant,  prefer cool and moist climates. |
| 3 | *Ulmus davidiana* | 2.1 | Deciduous broadleaf trees, moderately shade-tolerant,  exhibit strong adaptability to various climates. |
| 3 | *Tilia amurensis* | 3 | Deciduous broadleaf trees, shade-tolerant,  prefer cool and moist climates. |
| 3 | *Ulmus laciniata* | 3 | Deciduous broadleaf trees, moderately shade-tolerant,  exhibit strong adaptability to various climates. |
| 3 | *Acer triflorum* | 3.45 | Deciduous broadleaf trees, moderately shade-tolerant |
| 3 | *Acer mandshuricum* | 3.45 | Deciduous broadleaf trees, moderately shade-tolerant. |
| 3 | *Acer tegmentosum* | 3.45 | Deciduous broadleaf trees, moderately shade-tolerant. |
| 3 | *Acer pictum* | 4 | Deciduous broadleaf trees, moderately shade-tolerant. |
| 3 | *Carpinus cordata* | 1.98 | Deciduous trees, preferring light and moist conditions. |
| 2 | *Styphnolobium japonicum* | 2 | Deciduous broadleaf small or large trees, preferring light. |
| 2 | *Phellodendron amurense* | 1.67 | Deciduous broadleaf trees, preferring light,  tolerant to severe cold, suitable for cold and humid climates. |
| 2 | *Malus baccata* | 2 | Deciduous broadleaf small or large trees, preferring light. |
| 2 | *Syringa reticulata* | 1.35 | Deciduous broadleaf small or large trees, preferring light. |
| 2 | *Betula costata* | 1.62 | Deciduous broadleaf trees, preferring moderate light,  favoring cold and humid conditions. |
| 1 | *Fraxinus mandshurica* | 2 | Deciduous broadleaf large trees, moderately preferring light,  fast-growing species with a long lifespan. |
| 1 | *J**uglans mandshurica* | 1.74 | Deciduous broadleaf trees, preferring light, strong cold resistance, and long lifespan. |
| 1 | *Populus ussuriensis* | 1.6 | Deciduous broadleaf trees, preferring light, fast-growing species, tolerant to cold and drought. |
| 1 | *Quercus mongolica* | 2.1 | Deciduous broadleaf trees, preferring light, favoring cool climates. |
| 1 | *Betula platyphylla* | 1.25 | Deciduous broadleaf trees, preferring light, fast-growing species. |

Table S2. Sample size distribution of the three functional groups across the four thinning treatments.

| **Functional group** | **Control (0%)** | **Low (20%)** | **Moderate (40%)** | **High (60%)** |
| --- | --- | --- | --- | --- |
| Cluster 1 | 110 | 132 | 127 | 72 |
| Cluster 2 | 39 | 30 | 33 | 13 |
| Cluster 3 | 481 | 443 | 367 | 258 |


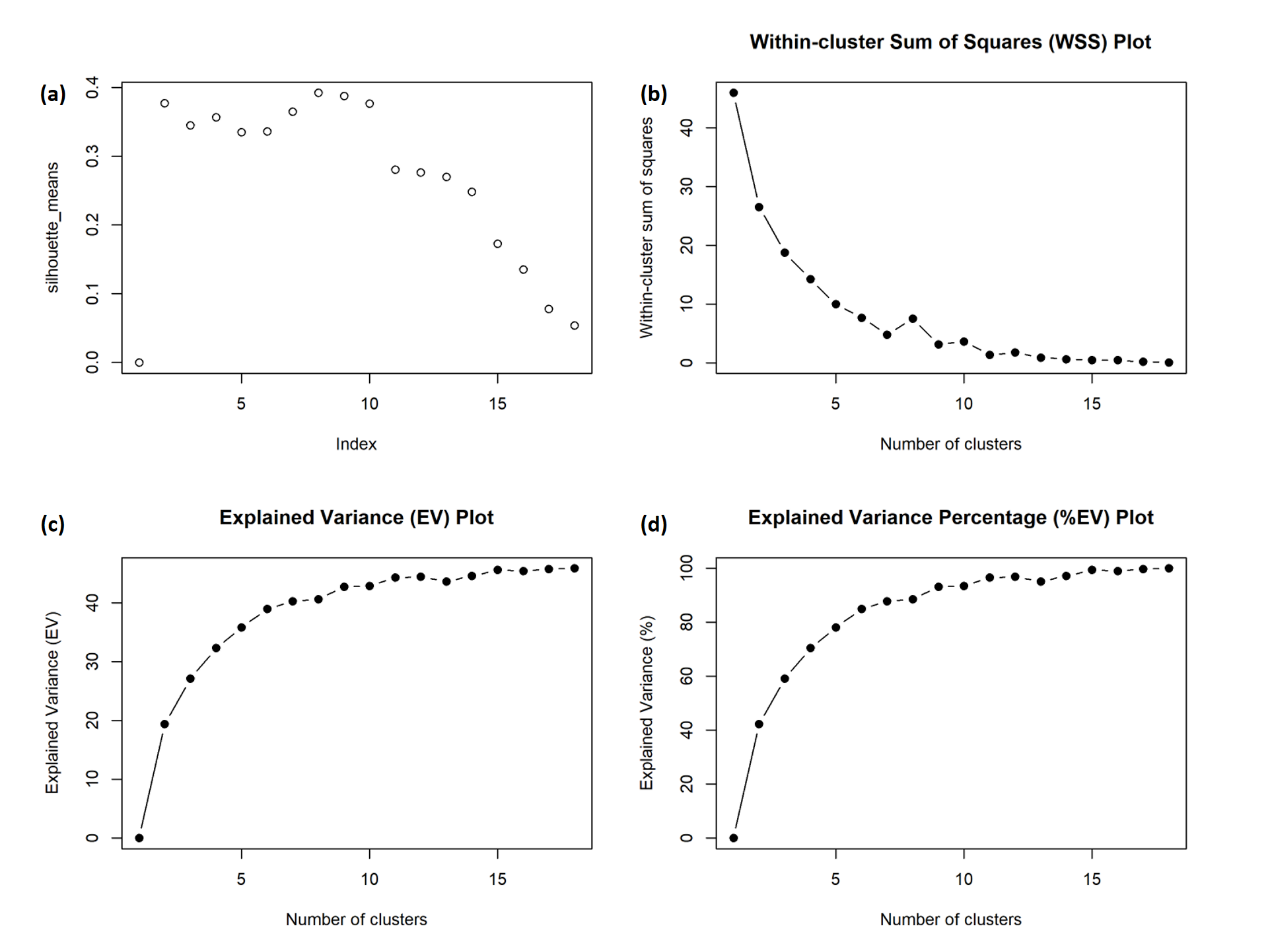


**Fig. S1 Average silhouette width (A), within-cluster sum of squares plots (B), explained variance plot (C), and explained variance percentage plot (D).**


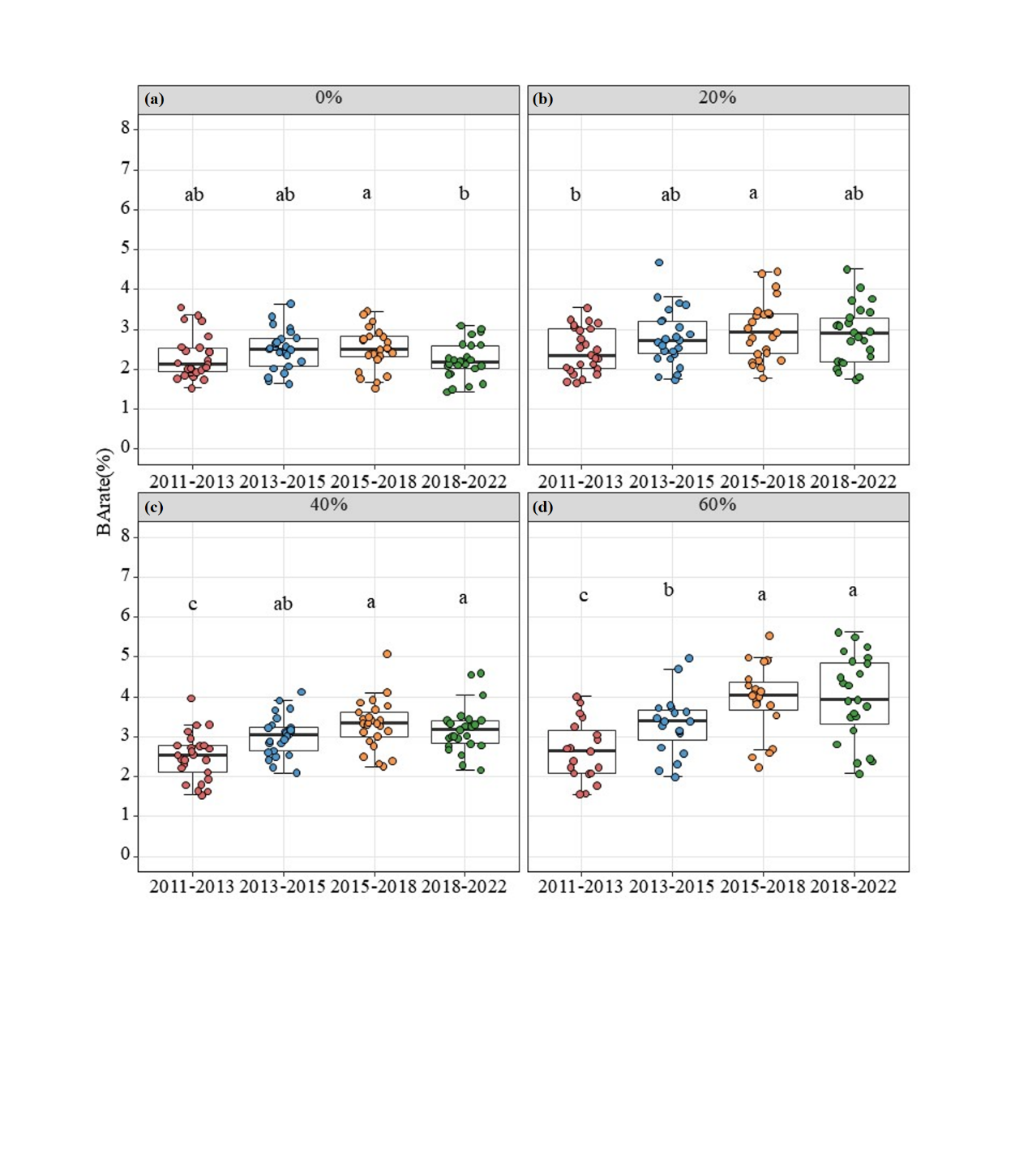


**Fig.S2** Changes in tree BA rate (Basal Area rate) during each recovery period under different thinning intensities. Different lowercase letters indicate significant differences among treatments within the same species group at *p* < 0.05.


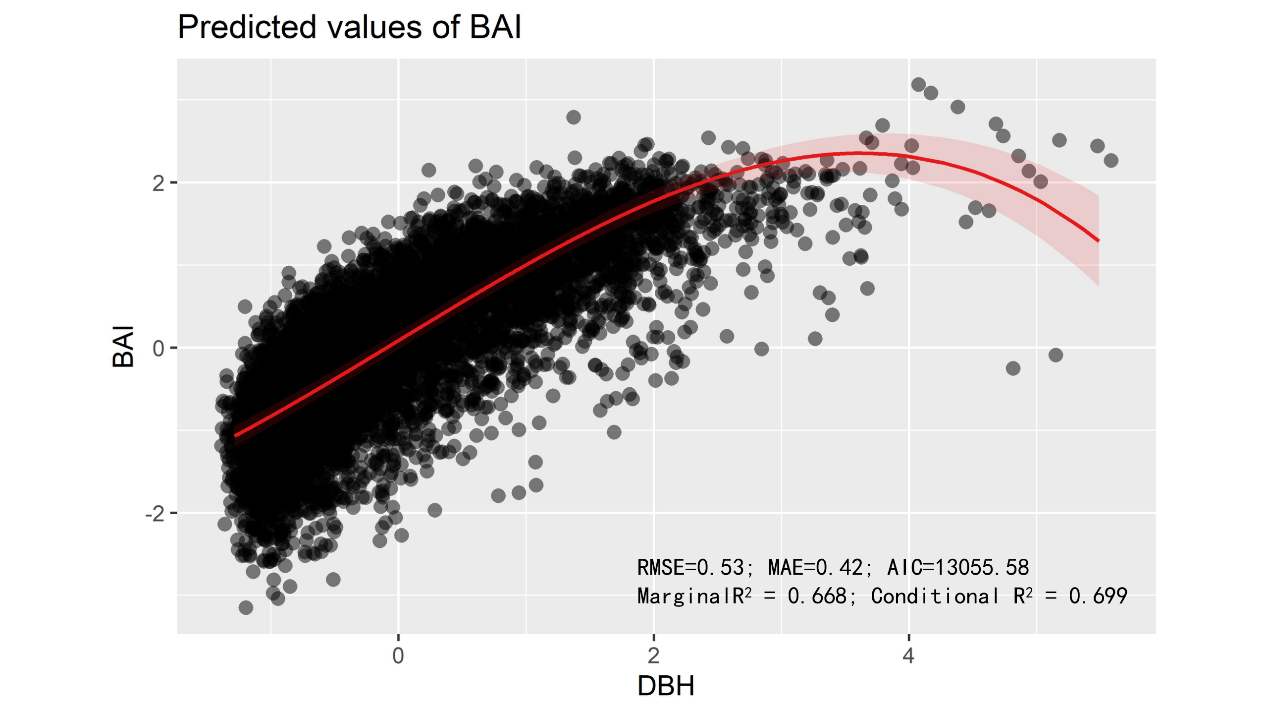


**Fig. S3** The predictive performance of mixed effect models. The black dots represent the points corresponding to the DBH and basal area increment for each tree, while the red line represents the fitted model. RMSE is the square root of the average of the squared differences between the predicted values and the actual values. MAE is the average of the absolute differences between the predicted values and the actual values. AIC is Akaike Information Criterion. Marginal R^2^ represents the proportion of variance explained by the fixed effects in a mixed-effects model. Conditional R^2^ represents the proportion of variance explained by both the fixed and random effects in a mixed-effects model.


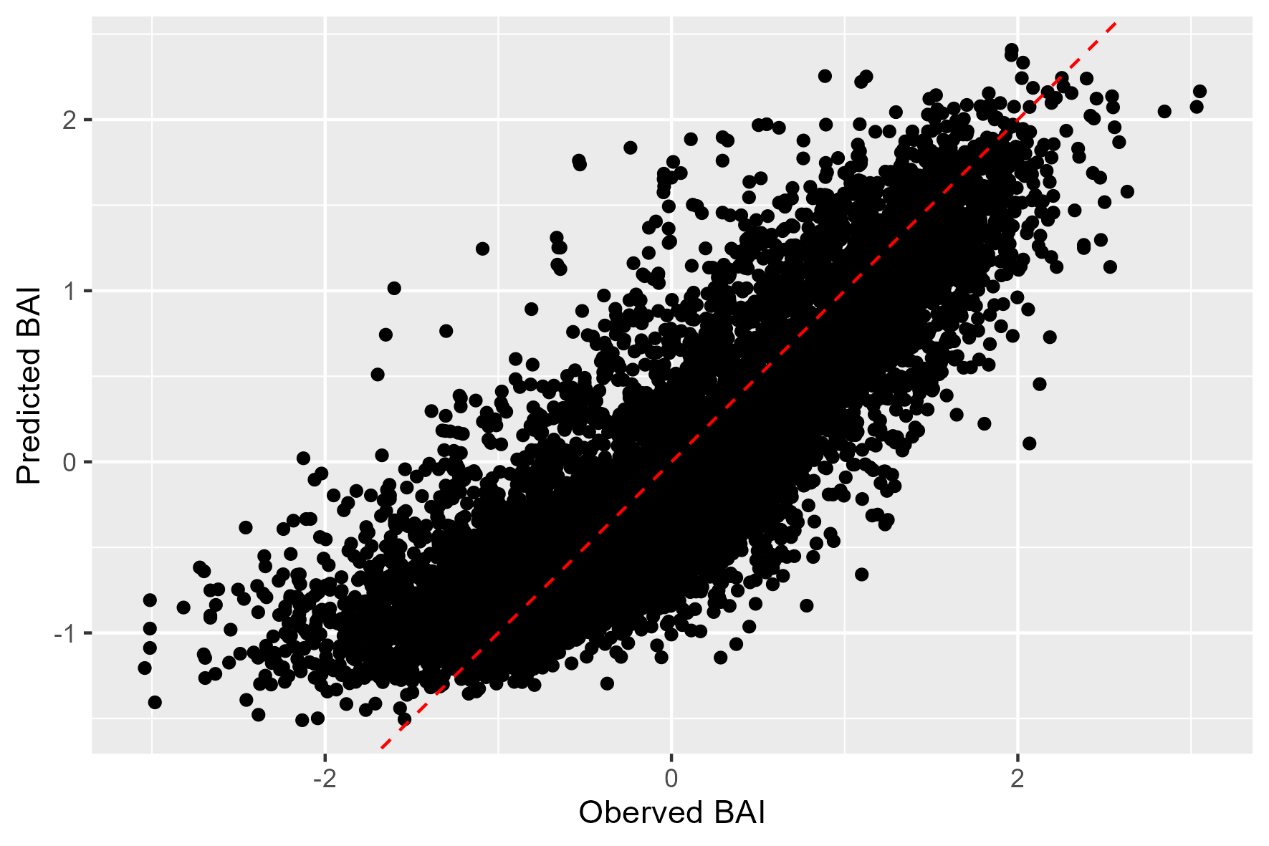


**Fig. S4** Comparison of observed and predicted basal area increment (BAI). The black dots represent the observed values of basal area increment corresponding to the model's predicted values, while the red dashed line represents the diagonal. The closer the black dots are to the red dashed line, the better the model's fit.


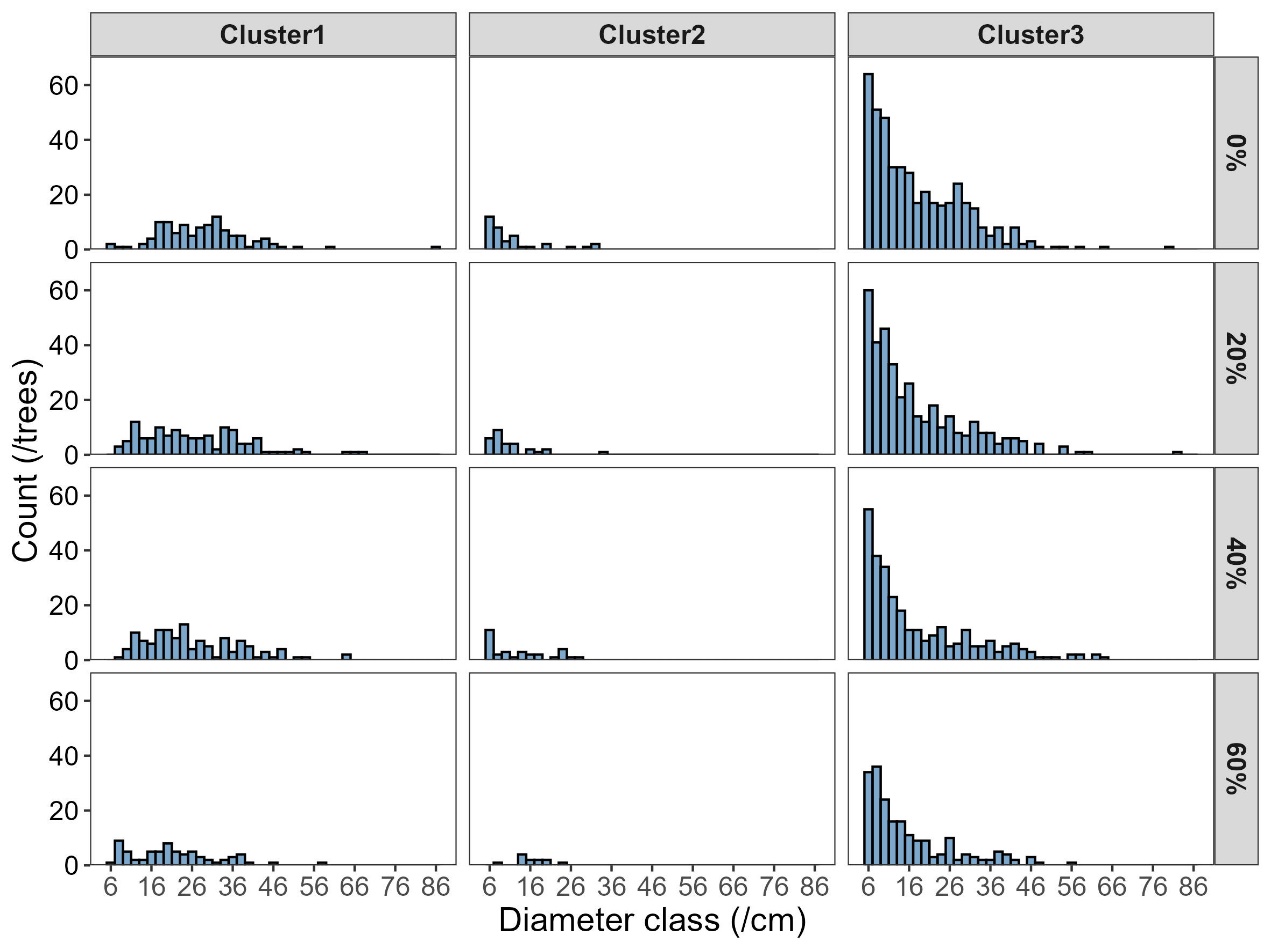


**Fig.S5 Diameter distribution chart for each group under different thinning intensities.**
